# Anatomical and intraoperative predictors of surgical complications after distal hypospadias repair with foreskin reconstruction: a prospective study

**DOI:** 10.1007/s00345-025-06082-6

**Published:** 2025-12-04

**Authors:** Maria Escolino, Claudia Di Mento, Francesca Carraturo, Annalisa Chiodi, Fulvia Del Conte, Giovanni Esposito, Vincenzo Coppola, Maria Sofia Caracò, Benedetta Cesaro, Maria Luisa Pirone, Ciro Esposito

**Affiliations:** 1 Division of Pediatric Surgery, Federico II University Hospital, Via Pansini, 5, Naples, 80131 Italy; 2 CEINGE Advanced Biotechnologies, Naples, Italy

**Keywords:** Hypospadias, Urethroplasty, Anatomy, Complications, Risk factors, Children

## Abstract

**Purpose:**

This study aimed to evaluate anatomical and intraoperative risk factors predicting complications following distal hypospadias repair with foreskin reconstruction in pediatric patients.

**Methods:**

A prospective analysis was conducted on all patients with distal hypospadias undergoing tubularized incised plate urethroplasty (TIPU) with foreskin reconstruction over a 2-year period. Intraoperative measurements—stretched penile length, glans width, glans groove width, meatal position, ischemic time and preputial symmetry—were recorded. Postoperative complications and reoperations were analyzed through multivariate logistic regression to determine independent predictors.

**Results:**

The patient cohort included 71 operated boys. Median patient age was 2.4 years (range 1-9.1), and median follow-up was 12.8 months. The most frequent complications were foreskin dehiscence (12.7%), non-retractile foreskin (12.7%), meatal/urethral stenosis (11.3%), and urethrocutaneous fistula (7.0%). Prolonged ischemic time (> 60 min) independently predicted foreskin dehiscence (*p* = 0.03) and reoperation (*p* = 0.003). Shorter PL (≤ 3 cm) was associated with higher stenosis rates, while more proximal distal meatal position (subcoronal/midpenile) increased the risk of foreskin dehiscence (*p* = 0.0006), fistula (*p* = 0.007), and reoperation (*p* = 0.02). Age > 2 years significantly predicted meatal/urethral stenosis (*p* = 0.01) and reoperation (*p* = 0.001). Preputial asymmetry correlated with foreskin dehiscence (*p* = 0.006), whereas glans width and groove width showed no significant associations (*p* > 0.05).

**Conclusion:**

Prolonged ischemic time, shorter penile length, more proximal distal meatal position, asymmetric prepuce and older age at surgery are independent predictors of postoperative complications and reoperations after distal hypospadias repair with foreskin reconstruction. Careful preoperative assessment, minimizing intraoperative ischemic duration, and considering earlier surgery before 2 years of age may improve both functional and cosmetic outcomes.

## Introduction

Hypospadias is one of the most common congenital anomalies of the male genitalia, with an incidence ranging between 1:200 and 1:300 live male births [1, 2]. The lack of a standardized classification system for hypospadias makes it difficult to include anatomical variations among patients and assess surgical outcomes effectively [3–5]. Surgical correction of hypospadias has evolved over the past decades. Tubularized incised plate urethroplasty (TIPU) is commonly employed for distal hypospadias repair in many centers and is well-documented in the literature [6–8]. Foreskin reconstruction has also gained attention in selected populations, although its adoption remains variable worldwide [9, 10]. Foreskin reconstruction has an 8% risk of specific complications, such as dehiscence or secondary phimosis requiring circumcision. However, it does not appear to increase the likelihood of urethroplasty complications or affect the overall reoperation rate for hypospadias repair [11, 12]. Despite advances in surgical methods and perioperative care, postoperative complications remain a concern [13–15].

Identifying predictive risk factors for postoperative complications is crucial in optimizing surgical planning, improving outcomes, and reducing reoperation rates. Several studies have already investigated various anatomical and intraoperative factors that may influence surgical success, without drawing any definitive evidence [16–21].

We hypothesized that specific anatomical and intraoperative parameters would be predictive of postoperative complications following distal hypospadias repair.

This study aimed to provide a comprehensive evaluation of preoperative anatomical characteristics and intraoperative factors that may correlate with surgical complications to improve risk stratification, patient selection and surgical techniques.

## Materials and methods

### Study design and population

We conducted a prospective cohort study over a 2-year period (January 2023-January 2025) at a tertiary pediatric urology center. All patients included in the study had a confirmed diagnosis of distal hypospadias with penile curvature ≤ 30° and were scheduled to undergo tubularized incised plate urethroplasty (TIPU) with foreskin reconstruction. Patients with distal (glanular) or proximal hypospadias, as well as those with severe penile curvature (> 30°) requiring staged correction, were excluded from the study. Cases presenting severe ventral skin deficiency who underwent circumcision, as well as those circumcised due to the surgeon’s decision or parental request, were excluded. Additionally, patients who had undergone preoperative hormonal therapy (either topical or parenteral testosterone) were also excluded, because such treatment can modify penile and preputial anatomy by increasing penile length, glans width, and vascularization, potentially influencing intraoperative measurements and postoperative outcomes. To avoid this confounding effect, only patients without any prior hormonal stimulation were included.

The study received appropriate Institute Review Board (IRB) approval.

## Intraoperative assessment

**Fig. 1:**
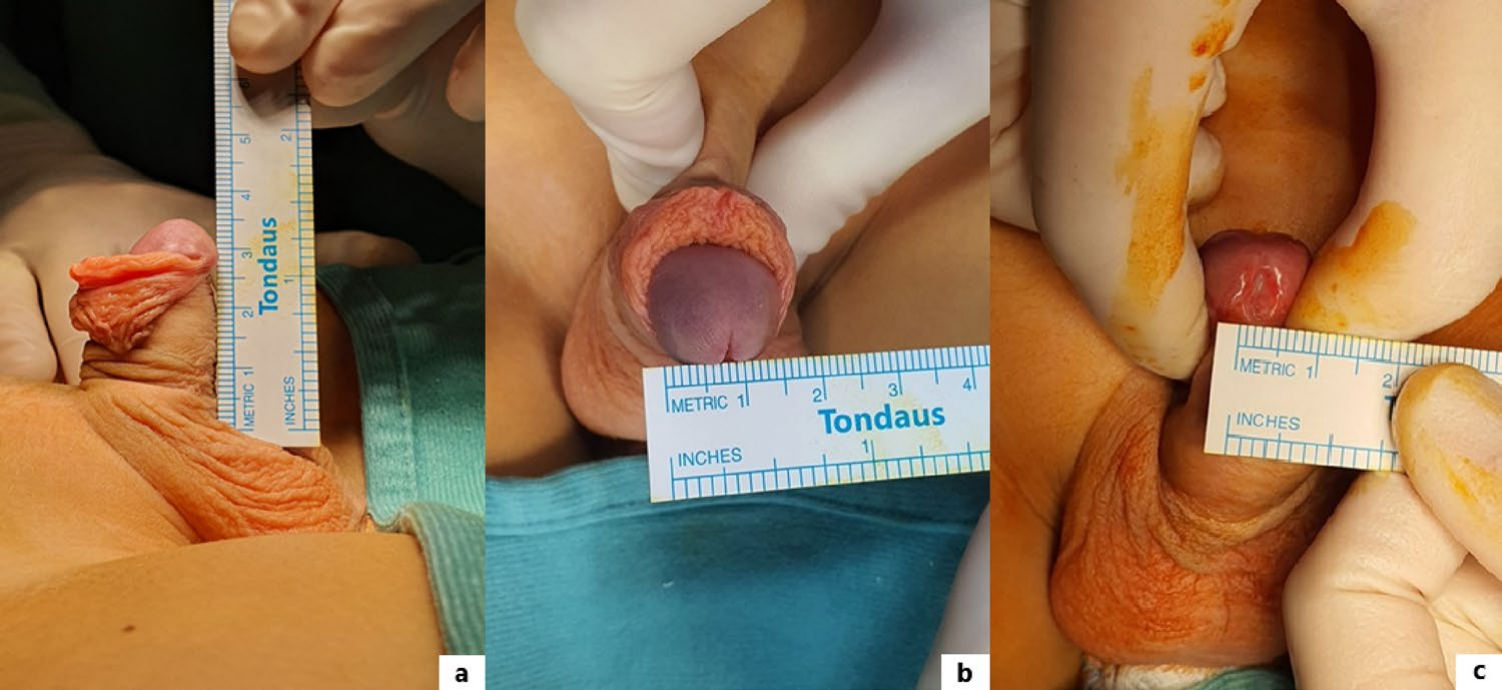
Intraoperative measurements: stretched penis length (PL) (**a**); glans width (GW) (**b**); glans groove width (GGW) (**c**)

Anatomical measurements were taken intraoperatively while the patient was under general anesthesia before any skin marking or surgical incision. Measurements were performed by using a ruler and recorded by two experienced pediatric surgeons to ensure consistency (Fig. 1). Data were recorded in an electronic database to ensure standardization and facilitate future analysis. The measured parameters included stretched penile length (PL), expressed in centimeters (cm) (Fig. 1a); glans width (GW), measured at the widest ventral point of the glans, expressed in millimeters (mm) (Fig. 1b); glans groove width (GGW), measured at its narrowest point between the lateral edges, expressed in mm (Fig. 1c); penile curvature, recorded binarily as yes/no; penile torsion, recorded binarily as yes/no; meatal position, categorized as coronal, subcoronal or midpenile; and ischemic time, defined as the duration of the indwelling tourniquet in minutes. Tourniquet time was continuous and measured from application to release. In addition, we assessed preputial asymmetry, not as a quantitative variable but as a qualitative intraoperative finding. The prepuce was classified as symmetric or asymmetric, based on the surgeon’s direct evaluation of the lateral wings and the presence of the “Ombredanne eyes” pattern.

## Surgical technique

All procedures were performed under general anesthesia with caudal epidural analgesia to provide optimal perioperative pain control. Two senior pediatric surgeons performed all surgeries to minimize variability.

The surgical steps of the TIPU technique included:

- Degloving the penis: a ventral incision was made along the junction between the inner and outer prepuce, extending around the meatus, followed by ventral degloving down to the penoscrotal junction. A soft silicone tourniquet was applied at the penoscrotal junction immediately before penile degloving and maintained until completion of urethroplasty.
- Artificial erection test: a 21G butterfly needle was used to inject normal saline into the glans.
- Curvature assessment: The angle of curvature was measured using either a small goniometer or visual inspection. If the penis was straight or had a curvature of less than 30°, a single-stage repair was performed.
- Midline incision and mobilization: A deep incision was made along the urethral plate and in the middle, and the glans wings were mobilized. The dissection was extended to the surface of the corpora cavernosa and continued laterally to facilitate midline approximation without tension.
- Urethral tubularization: The urethral plate was tubularized using subepithelial continuous 6−0 polydioxanone suture.
- Dartos flap reinforcement: A well-vascularized dartos flap was mobilized and placed over the neourethra to prevent fistula formation.
- Glansplasty and meatal reconstruction: Glans wings were approximated in 2 layers with subepithelial 6 − 0 and 5 − 0 polydioxanone suture to ensure a functional and natural-looking neomeatus.
- Preputioplasty: foreskin reconstruction was performed in 3 layers (inner prepuce, dartos and skin) with interrupted 6−0 polydioxanone sutures.

## Postoperative care and follow-up

An 8- or 10-F urethral catheter and a tubular ozonated oil inside-coated device were placed for 5–7 days, according to our standard hypospadias dressing protocol [22]. Parents were educated on proper postoperative care, including wound management, hygiene, and identifying early signs of complications. Patients were advised to apply ozonated oil externally over the reconstructed foreskin without retraction for 3–4 weeks following the dressing removal. Additionally, parents were instructed to avoid retracting the foreskin for at least 6–8 weeks after surgery. Follow-up visits were conducted on 7, 14, 30, 90, 180, and 360 postoperative days. Evaluations included physical examination for signs of healing complications, assessment of urinary function and cosmesis. Complications were assessed at each follow-up visit using a checklist. Meatal stenosis was defined by a narrowed meatus (< 6Fr) associated with obstructive voiding symptoms. Other complications included urethrocutaneous fistula (UCF), urethroplasty breakdown, glans dehiscence, foreskin dehiscence, and non-retractile foreskin. Postoperative complications were graded according to Clavien-Dindo classification [23]. Patients requiring additional interventions were scheduled for redo procedures as needed.

### Statistical analysis

A comprehensive statistical analysis was performed using IBM SPSS Statistics, version 27. Initially, descriptive statistics were applied to summarize the patient characteristics and complication rates. Categorical variables were expressed as percentages and compared using the Chi-square test or Fisher’s exact test. Continuous variables were reported as median with interquartile range (IQR). To compare these continuous variables between groups, the Mann-Whitney U test was employed for non-parametric distributions. To determine independent risk factors for postoperative complications, multivariate logistic regression models were constructed. The multivariate logistic regression analysis of complications and reoperations was performed between the risk factor groups: short penis (PL ≤ 3 cm) vs. long penis (PL >3 cm), large glans (GW >10 mm) vs. small glans (GW ≤ 10 mm), wide urethral plate (GGW >5 mm) vs. narrow urethral plate (GGW ≤ 5 mm), meatal position distal (coronal) vs. proximal (subcoronal/midpenile), age at surgery ≤ 2 years vs. >2 years, and ischemic time ≤ 60 min vs. >60 min. A 60-minute cutoff for ischemic time was selected based on published data reporting compromised penile perfusion beyond this threshold [24, 25] and on the median ischemic time observed in our cohort, ensuring a balanced and clinically relevant comparison. The adjusted odds ratios (ORs) were reported with 95% confidence intervals (CIs), and statistical significance was set at *p* < 0.05.

## Results

The patient cohort included 71 boys with distal hypospadias who underwent TIPU and preputioplasty over the study period. The median patient age at time of surgical repair was 2.4 years (range 1–9.1). Meatal position was coronal in 51 patients (71.8%), subcoronal in 11 (15.5%), and midpenile in 9 (12.7%). Intraoperative measurements revealed mean PL of 3.23 cm (range 2.0-5.8), GW of 13.5 mm (range 8–22), GGW of 6.08 mm (range 2.0–13.0), and ischemic time of 68.17 min (range 40–120). The distribution of anatomical measurements showed that 11/71 patients (15.5%) had GW ≤ 10 mm, while 60/71 (84.5%) had GW > 10 mm.

At a median follow-up of 12.8 months (range 6–24), postoperative complications were recorded in multiple domains. Foreskin dehiscence occurred in 9/71 patients (12.7%), non-retractile foreskin in 9/71 (12.7%), meatal/urethral stenosis in 8/71 (11.3%), UCF in 5/71 (7.0%), and glans dehiscence in 1/71 (1.4%). Eleven patients required 13 reoperations (18.3%) (Clavien–Dindo grade IIIb). Among the reoperated patients, 5/71 (7.0%) underwent urethral calibration under anesthesia for meatal/urethral stenosis. One of these patients required 3 urethral calibrations to solve the urehtral stenosis. Four/71 patients (5.6%) underwent UCF closure with circumcision, 1/71 (1.4%) with glans dehiscence underwent redo-TIPU with circumcision, and 1/71 (1.4%) with foreskin dehiscence underwent circumcision. Additionally, 9/71 (12.7%) patients who developed non-retractile foreskin were successfully treated with topical corticosteroids (Clavien–Dindo grade II). These complications were identified at a median follow-up of 4.8 months (range 1–11).

Patient characteristics, intraoperative measurements, and postoperative outcomes are summarized in Table 1.

**Table 1:** Patient characteristics, intraoperative measurements, and postoperative outcomes

Patient characteristics	
Number of patients, n	71
Median age, years (range)	2.4 (1.0–9.1)
Meatal position: 1. Coronal, n (%) 2. Subcoronal, n (%) 3. Midpenile, n (%)	51 (71.8) 11 (15.5) 9 (12.7)
Median follow-up, months (range)	12.8 (6–24)

Intraoperative measurements	Mean (range)	Standard deviation (SD)
Stretched penis length (PL), (cm)	3.23 (2.0–5.8)	0.74
Glans width (GW), (mm)	13.5 (8–22)	3.11
Glans groove width (GGW), (mm)	6.08 (2.0–13.0)	2.42
Ischemic time, (minutes)	68.17 (40–120)	16.69
Postoperative outcomes		
Complications		
1. Meatal/urethral stenosis, n (%)	8 (11.3)	
2. Foreskin dehiscence, n (%)	9 (12.7)	
3. Urethrocutaneous fistula, n (%)	5 (7.0)	
4. Glans dehiscence, n (%)	1 (1.4)	
5. Non retractile foreskin, n (%)	9 (12.7)	
Reoperations, n (%)	13 (18.3)	

The multivariate logistic regression analysis identified ischemic time exceeding 60 min as an independent predictor of foreskin dehiscence (*p* = 0.03) and reoperation (*p* = 0.003). PL ≤ 3 cm emerged as an independent predictor of meatal/urethral stenosis. More proximal meatal position (subcoronal/midpenile) was associated with significantly higher risk of foreskin dehiscence (*p* = 0.0006), UCF (*p* = 0.007) and reoperation (*p* = 0.02). Patient age > 2 years were found to have significantly higher risk of meatal/urethral stenosis (*p* = 0.01) and reoperation (*p* = 0.001). No statistically significant correlations were observed between glans width, glans groove width and the evaluated complications (*p* > 0.05). Conversely, preputial asymmetry was found to be significantly associated with a higher risk of foreskin dehiscence compared to patients with a symmetric prepuce (*p* = 0.006).

A summary of multivariate logistic regression findings is presented in Table 2.

**Table 2:** Multivariate logistic regression analysis

Foreskin dehiscence	OR	95% CI lower	95% CI upper	Chi-square	*p* value
PL ≤ 3 cm vs. > 3 cm	0.75	0.18	3.06	0.16	0.69
GW ≤ 10 mm vs. > 10 mm	0.65	0.07	5.79	0.15	0.7
GGW ≤ 5 mm vs. > 5 mm	0.75	0.18	3.06	0.16	0.69
Ischemic time ≤ 60 min vs. > 60 min	0.13	0.02	1.13	4.44	**0.03**
Meatal position	0.06	0.01	0.47	11.67	**0.0006**
Age ≤ 2y vs. > 2y	1.79	0.34	9.4	0.49	0.49
Asymmetric prepuce	6.86	1.52	30.99	7.55	**0.006**

Phimosis	OR	95% CI lower	95% CI upper	Chi-square	*p* value
PL ≤ 3 cm vs. > 3 cm	2.13	0.49	9.30	1.05	0.31
GW ≤ 10 mm vs. > 10 mm	1.68	0.3	9.42	0.36	0.55
GGW ≤ 5 mm vs. > 5 mm	0.75	0.18	3.06	0.16	0.69
Ischemic time ≤ 60 min vs. > 60 min	0.61	0.14	2.65	0.45	0.5
Meatal position	8.57	0.47	155.34	2.93	0.09
Age ≤ 2y vs. > 2y	4.4	0.52	37.51	2.13	0.14
Asymmetric prepuce	2.3	0.55	9.64	1.35	0.25

Urethrocutaneous fistula	OR	95% CI lower	95% CI upper	Chi-square	*p* value
PL ≤ 3 cm vs. > 3 cm	0.63	0.10	4.0	0.25	0.62
GW ≤ 10 mm vs. > 10 mm	0.5	0.03	9.83	0.22	0.64
GGW ≤ 5 mm vs. > 5 mm	0.63	0.10	4.0	0.25	0.62
Ischemic time ≤ 60 min vs. > 60 min	0.11	0.01	2.15	2.95	0.09
Meatal position	0.08	0.01	0.77	7.14	**0.007**
Age ≤ 2y vs. > 2y	0.29	0.04	1.87	1.87	0.17
Asymmetric prepuce	1.78	0.27	11.53	0.37	0.54

Glans dehiscence	OR	95% CI lower	95% CI upper	Chi-square	*p* value
PL ≤ 3 cm vs. > 3 cm	2.0	0.06	61.57	0.16	0.69
GW ≤ 10 mm vs. > 10 mm	12.0	0.38	381.88	3.09	0.08
GGW ≤ 5 mm vs. > 5 mm	2.0	0.06	61.57	0.16	0.69
Ischemic time ≤ 60 min vs. > 60 min	0.64	0.02	19.70	0.07	0.80
Meatal position	0.19	0.01	5.90	1.11	0.29
Age ≤ 2y vs. > 2y	0.23	0.01	7.09	0.84	0.36
Asymmetric prepuce	1.25	0.04	38.76	0.02	0.90

Meatal/urehtral stenosis	OR	95% CI lower	95% CI upper	Chi-square	*p* value
PL ≤ 3 cm vs. > 3 cm	8.21	0.95	70.67	4.88	**0.03**
GW ≤ 10 mm vs. > 10 mm	4.13	0.82	20.68	3.33	0.07
GGW ≤ 5 mm vs. > 5 mm	0.97	0.22	4.22	0.002	0.97
Ischemic time ≤ 60 min vs. > 60 min	0.16	0.02	1.35	3.56	0.06
Meatal position	0.62	0.13	2.86	0.39	0.53
Age ≤ 2y vs. > 2y	0.12	0.02	0.67	7.47	**0.01**
Asymmetric prepuce	2.94	0.66	13.13	2.12	0.15

Reoperation	OR	95% CI lower	95% CI upper	Chi-square	*p* value
PL ≤ 3 cm vs. > 3 cm	1.17	0.35	3.9	0.06	0.80
GW ≤ 10 mm vs. > 10 mm	3.24	0.78	13.37	2.84	0.09
GGW ≤ 5 mm vs. > 5 mm	0.36	0.10	1.31	2.53	0.11
Ischemic time ≤ 60 min vs. > 60 min	0.08	0.01	0.62	8.68	**0.003**
Meatal position	0.25	0.07	0.87	5.18	**0.02**
Age ≤ 2y vs. > 2y	0.14	0.04	0.53	9.86	**0.001**
Asymmetric prepuce	1.17	0.31	4.33	0.05	0.82

*PL* penis length, *GW* glans width, *GGW* glans groove width

## Discussion

Despite the current advancements in surgical techniques, hypospadias repair continues to have a significant rate of postoperative complications. The identification of risk factors for these complications is still being explored. Anatomical measurements taken during distal hypospadias repair can have predictive value on surgical outcomes [26].

In this prospective cohort of 71 patients undergoing TIPU with foreskin reconstruction, we identified several anatomical and intra-operative factors that significantly predicted adverse outcomes, providing useful insights for surgical planning and patient counseling. One of the most significant observations was the strong correlation between prolonged ischemic time (>60 min) and increased risk of foreskin dehiscence and reoperation. This finding underlines the critical importance of minimizing surgical time and ensuring adequate tissue perfusion during the procedure to enhance healing outcomes and reduce postoperative morbidity. Previous studies have shown that tourniquet application exceeding approximately 60 min may impair penile microcirculation and increase the risk of wound-healing complications during hypospadias repair [24, 25]. In our clinical practice, the tourniquet was applied before penile degloving and maintained until completion of urethroplasty. Therefore, both the penile skin and prepuce were included within the ischemic field during the entire degloving and urethroplasty phases. However, as degloving was performed rapidly (within 5–7 min), the largest portion of ischemic exposure corresponded to the time required for urethroplasty and glans reconstruction. Other authors proposed using noninvasive erection tests to replicate the body’s normal erection process during surgery without requiring repeated punctures of the corpora cavernosa, thereby reducing the risks and complications associated with the artificial erection test [27, 28].

In addition to ischemic time, we found that preputial asymmetry also influenced surgical outcomes. Patients with an asymmetric prepuce, characterized by unequal lateral wings or presence of Ombredanne eyes, showed a significantly higher risk of foreskin dehiscence. This finding highlights the relevance of preputial morphology assessment in preoperative planning and patient selection for foreskin reconstruction.

Interestingly, shorter penile length (≤ 3 cm) was identified as an independent predictor of meatal/urethral stenosis. We think that this correlation may be linked to reduced tissue availability for a tension-free reconstruction, thus increasing the risk of suture line narrowing during the healing phase.

Meatal position had also a significant impact on surgical outcomes. Patients with subcoronal or midpenile meatus showed significantly higher risk of foreskin dehiscence, fistula formation and need for reoperation compared to those with coronal meatus. This is likely related to the longer urethral segment requiring reconstruction and increased technical complexity, predisposing to both urethral and preputial complications. These findings suggest that in more proximal distal variants, careful patient selection is crucial for foreskin reconstruction, with a critical balance between functional and cosmetic goals against potential risks.

Age at surgery also emerged as a relevant factor, with patients older than 2 years showing increased risk of meatal/urethral stenosis and reoperation. This result supports the importance of early surgical intervention, preferably before 2 years of age, to achieve optimal healing, better tissue handling, and reduce complication rates, in line with established pediatric urology guidelines [29].

Previous studies have reported that inadequate glans width can lead to insufficient tissue support, contributing to structural weakness at the surgical site [16, 19]. These studies demonstrated that small glans size, defined as glans width < 14 mm, was an independent risk factor for urethroplasty complications and the only significant predictor of both glans dehiscence and stenosis [16, 19]. Snodgrass et al. in 2011 [20] also identified glans anatomy as an important determinant of surgical success and emphasized the necessity of precise glans dissection to improve urethral plate stability and reduce dehiscence rates. Indeed, in contrast to previous studies, our analysis did not show a statistically significant association between glans size and occurrence of surgical complications (*p* >0.05). This may be due to differences in surgical technique, use of multilayer closures, careful glans dissection, and consistent postoperative care protocols in our center, which could decrease the impact of this variable.

The main limitations of this study include the relatively small single-center sample size and the absence of evaluation of other potentially relevant factors such as individual variability in wound healing, differences in surgical technique, and adherence to postoperative care. Another limitation is the relatively short follow-up period, which may underestimate the incidence of late complications such as urethral strictures that can appear beyond the first postoperative year. Nevertheless, all patients are being followed longitudinally, and extended follow-up data will be analyzed to confirm long-term outcomes and complication rates.

Despite these limitations, the clinical implications of this study are clear. First, reducing ischemic time during hypospadias repair should be prioritized as a critical surgical objective to minimize the risk of foreskin dehiscence and reoperation. The identification of non-modifiable anatomical parameters can guide patient selection and individualized surgical planning.

In conclusion, this study highlights the critical role of anatomical, individual, and intraoperative factors on the postoperative outcome of hypospadias surgery. Prolonged ischemic time, shorter penile length, more proximal distal meatal position, asymmetric prepuce and older age at surgery are independent predictors of postoperative complications and reoperations after distal hypospadias repair with foreskin reconstruction. Careful preoperative assessment, minimizing intraoperative ischemic duration, and considering earlier surgery before 2 years of age may improve both functional and cosmetic outcomes. Larger, multicenter studies focusing on a wider range of perioperative variables are warranted to validate these findings and develop strategies for patient selection, surgical planning, and complication prevention in distal hypospadias repair.

## Data Availability

No datasets were generated or analysed during the current study.
